# Migraine immune cell gene targets and their relationship to psychiatric disorders

**DOI:** 10.1186/s10194-026-02465-1

**Published:** 2026-07-25

**Authors:** Xin Mo, Dongren Sun, Fangfang Li, Danqi Wang, Yunjiao Deng, Yiwei Liao, Xiaosu Yang, Haiting Zhao

**Affiliations:** 1 Department of Neurosurgery, The Second People’s Hospital of Hunan Provincial (Hunan Provincial Brain Hospital), Changsha, 410011 China; 2 Department of Neurology, Hunan Provincial People’s Hospital, The First Affiliated Hospital of Hunan Normal University, Changsha, China; 3 Department of Neurosurgery, Xiangya Hospital, Central South University, Changsha, 410008 China; 4 FuRong Laboratory, Changsha, China; 5 Department of Neurology, Xiangya Hospital, Central South University, Changsha, 410008 China

**Keywords:** Migraine, Mendelian randomization, Single-cell eQTL, Psychiatric disorders, Drug–gene interactions

## Abstract

**Background:**

Migraine frequently co-occurs with psychiatric disorders, yet the immunogenetic mechanisms linking these conditions remain largely unexplored.

**Methods:**

Using cis-eQTL data from 28 immune cell subtypes (1,925 donors) and GWAS summary statistics for migraine and five psychiatric disorders, we performed single-cell transcriptome-wide Mendelian randomization, Bayesian colocalization, genetic correlation, and cross-disease pleiotropy analyses. Independent replication was performed using external datasets.

**Results:**

Migraine and its subtypes showed significant positive genetic correlations with all five psychiatric disorders (rg = 0.39–0.73). We identified 83 immune cell gene targets for migraine, 13 for migraine with aura, and 19 for migraine without aura. Among these, 6 targets showed shared associations with anxiety and 1 with depression. Three prioritized genes—HLA-A, CDK2AP1, and TTC24—demonstrated cross-disease pleiotropic effects. Notably, HLA-A in cDC1 exhibited discordant pleiotropy (protective for migraine with aura, risk for depression), with known drug–gene interactions involving antiepileptics and tricyclic antidepressants.

**Conclusions:**

These findings suggest that immune cell–specific genes, particularly HLA-A, CDK2AP1, and TTC24, may bridge migraine and psychiatric disorders, offering potential candidates for further investigation into shared immunogenetic mechanisms.

**Clinical trial number:**

Not applicable.

**Supplementary Information:**

The online version contains supplementary material available at 10.1186/s10194-026-02465-1.

## Introduction

Migraine is a highly prevalent primary headache disorder, affecting approximately 15% of the global population, with a lifetime incidence of 43% in women and 18% in men in the United States. Peak onset occurs at 20–24 years in women (18.2 per 1,000 person-years) and 15–19 years in men (6.2 per 1,000 person-years) [1, 2]. The disorder is classified into migraine without aura (the most common subtype) and migraine with aura (occurring in 25–30% of patients), with the lifetime prevalence of the former being roughly twice that of the latter [3]. Migraine exhibits substantial comorbidity with psychiatric disorders, showing a strong bidirectional relationship with depression and anxiety across all age groups [2]. Individuals with migraine have a 2.2- to 4.0-fold increased risk of depression, a 3.5- to 5.3-fold increased risk of generalized anxiety disorder, a 3.7-fold increased risk of panic disorder, and a 2.9- to 7.3-fold increased risk of bipolar disorder [4, 5]. A notable association with post-traumatic stress disorder has also been reported [4].

Shared immune dysregulation constitutes a common pathological substrate underlying both migraine and psychiatric disorders. These conditions are collectively characterised by heightened innate immune activation alongside a relative attenuation of adaptive immunity, as evidenced by neutrophilia, monocytosis, and perturbations in lymphocyte subsets—most notably a reduction in regulatory T cells (Tregs) and a Th1-polarised cytokine profile [6]. Transcriptomic analyses of peripheral blood have revealed 122 concordantly differentially expressed genes, including pentraxin 3 (PTX3) and haptoglobin (HP), both of which are upregulated in each condition and exhibit positive correlations with neutrophil and monocyte infiltration [7]. Pro-inflammatory mediators, including C-reactive protein (CRP), interleukin-1β (IL-1β), IL-6, and tumour necrosis factor-alpha (TNF-α), are significantly elevated in migraine patients, concomitant with diminished levels of the anti-inflammatory cytokine IL-10 [6]. Collectively, these observations indicate that peripheral immune dysregulation—particularly the overactivation of innate immunity and the disruption of the pro-inflammatory versus anti-inflammatory equilibrium—may represent a pivotal pathophysiological nexus linking migraine and psychiatric comorbidities, thereby furnishing a compelling rationale for the development of immune-targeted diagnostic and therapeutic interventions [6, 8].

Consequently, therapeutic strategies targeting specific immune cells have become a research focus. Anti-CGRP monoclonal antibodies have shown favorable efficacy and safety in patients with migraine comorbid with anxiety and depression, reducing monthly migraine days from 16 to 9 after six months, with a 50% response rate of 46% [9]. Other immunotherapies targeting the IL-1β/IL-1R1 axis, alongside potential agents such as hydroxychloroquine (targeting NELFCD) and bazedoxifene (targeting CDC42), are also under investigation [10, 11]. Despite these advances, clinical options remain limited and drug development is costly. Integrating genetic evidence into target discovery has become a critical pathway to improve development success rates, as genetically supported drug targets are more likely to succeed in later trials [12, 13]. Mendelian randomization (MR), which uses inherited genetic variants as instrumental variables to infer causal relationships, has emerged as a core strategy for identifying therapeutic targets [14, 15]. Its key advantage lies in mimicking drug action mechanisms, enabling pre-evaluation of therapeutic potential prior to large-scale randomized controlled trials and thereby enhancing research and development efficiency [16]. However, conventional MR relies on bulk-tissue expression quantitative trait locus (eQTL) data, which cannot resolve cell-type-specific heterogeneity and may overlook critical pathogenic pathways [17]. Recent breakthroughs in single-cell RNA sequencing have enabled the construction of cell-level eQTL maps, revealing cellular heterogeneity in genetic regulation and offering new avenues for investigating immune-cell-specific genetic signals [18, 19].

The present study employs single-cell transcriptome-wide Mendelian randomization (scTWMR), integrated with colocalization analysis, genetic correlation analysis, and cross-disease pleiotropy validation, to systematically evaluate immune-cell-specific genes jointly associated with migraine and psychiatric disorders—an approach not previously applied to this context. The primary objective is to identify such genes with shared genetic associations across both conditions. Secondary objectives include evaluating genetic correlations between migraine subtypes and psychiatric disorders, validating pleiotropic effects of prioritized targets, and exploring known drug–gene interactions as a hypothesis-generating framework.We hypothesize that cell-type-specific genetic signals will reveal distinct patterns of association with migraine and psychiatric disorders.

## Methods

### Overview

**Fig. 1:**
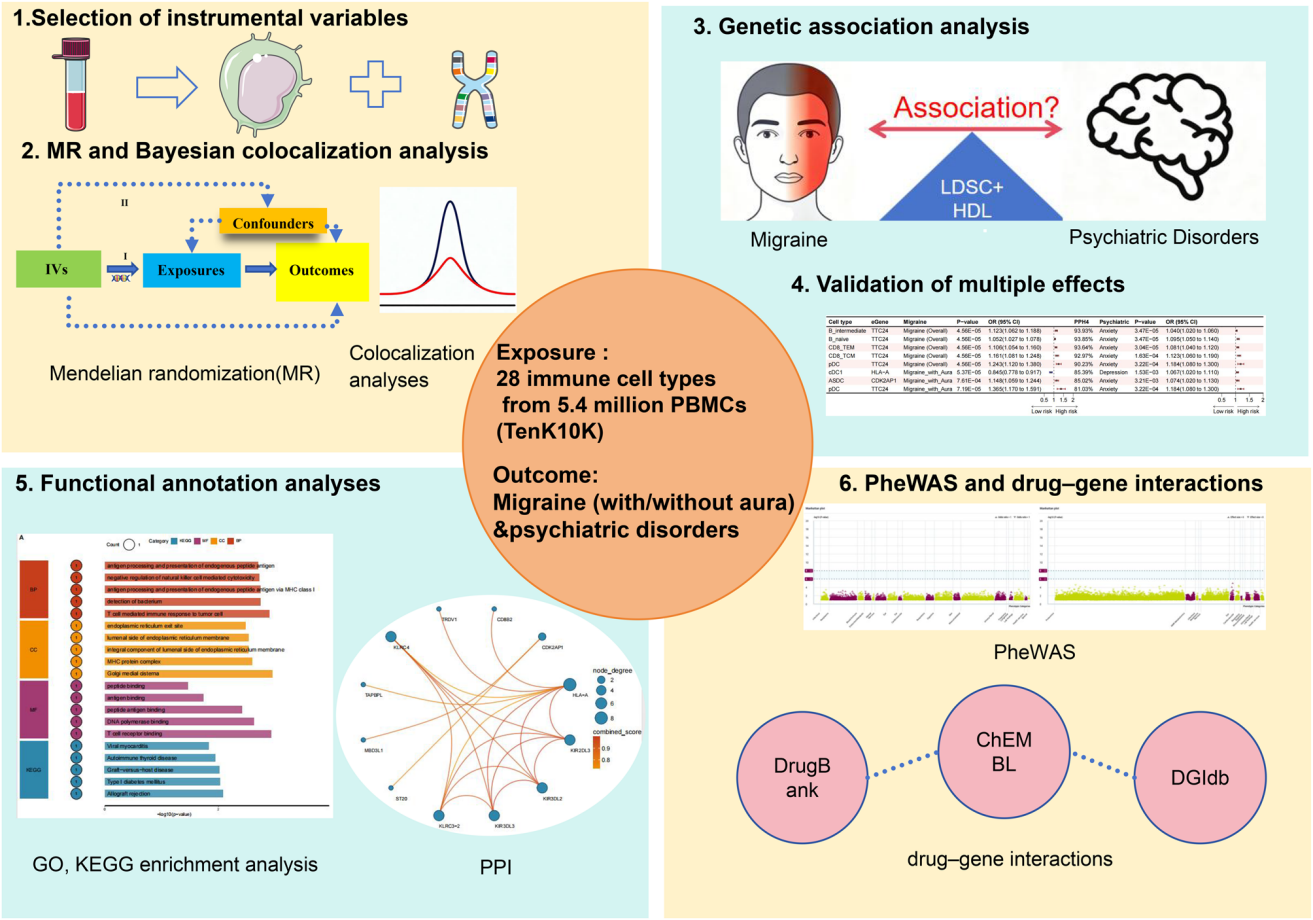
Study design for migraine immune cell gene targets and their relationship to psychiatric disorders

The technical workflow of this study is shown in Fig. 1. First, scTWMR combined with colocalization analysis was integrated to systematically identify the genetic associations of genes expressed in specific immune cell subtypes with migraine and its subtypes. Genetic correlations between migraine and common psychiatric disorders were assessed using linkage disequilibrium score regression (LDSC) and High-Definition Likelihood (HDL) methods, and cross-disease pleiotropy analysis was applied to explore the shared genetic associations of the aforementioned immune cell gene targets with migraine and psychiatric disorders. Subsequently, functional enrichment analysis and protein–protein interaction network construction were conducted to further investigate the biological processes and core regulatory pathways involving the identified gene sets. Finally, phenome-wide association analysis and database queries for known drug–gene interactions were performed to evaluate the safety and therapeutic potential of the prioritized targets. The entire study protocol strictly adhered to the recommendations outlined in the STROBE-MR guideline [20].

### Selection of instrumental variables

In this study, we utilized single-cell eQTL data from the TenK10K project to comprehensively screen cis-expression quantitative trait loci (cis-eQTLs) for constructing instrumental variables suitable for Mendelian randomization analysis [21]. The dataset comprises 1,925 individuals of European ancestry and over 5.4 million peripheral blood mononuclear cells, classified into 28 cell subtypes based on transcriptomic profiles. For each gene–cell type combination, candidate cis-eQTLs were identified within a 100 kb window flanking the gene’s transcription start site. Validation data were derived from the OneK1K single-cell eQTL dataset [18], which includes 982 healthy individuals of Nordic ancestry, yielding 1,267,798 peripheral blood mononuclear cells assigned to 14 distinct cell subtypes based on transcriptomic expression profiles.

Instrumental variable selection followed a multi-stage quality control procedure based on established methodologies [22]. Cis-eQTLs reaching genome-wide significance (*p* < 5 × 10^− 8^) were first selected. Single nucleotide polymorphisms (SNPs) were then clumped using a stringent linkage disequilibrium threshold (r^2^ < 0.001) based on the 1000 Genomes Project European reference panel to ensure statistical independence. During subsequent data harmonization, SNPs with ambiguous allele information or inconsistent strand orientation were excluded. To assess instrument reliability, we calculated the F-statistic for each SNP and excluded those with F < 10; the resulting F-statistics ranged from 29.74 to 997.62, effectively minimizing estimation bias due to weak instruments [23].

### Outcome data source

The migraine data (encompassing its subtypes) and the psychiatric disorder data—comprising anxiety disorder (ANX), depression, bipolar disorder (BIP), obsessive-compulsive disorder (OCD), and post-traumatic stress disorder (PTSD)—were obtained from the European-ancestry summary statistics of the FinnGen consortium R12 release [24]. The migraine cohort comprised 26,894 cases and 374,605 controls, of which the migraine-with-aura subtype included 11,757 cases and the migraine-without-aura subtype included 9,690 cases. ANX comprised 56,552 cases and 362,304 controls; depression comprised 59,333 cases and 434,831 controls; BIP comprised 8,946 cases and 434,831 controls; OCD comprised 2,813 cases and 444,414 controls; and PTSD comprised 3,444 cases and 444,414 controls. Validation data for migraine were derived from a genome-wide association meta-analysis conducted by Wema et al. [25], which included 135,458 cases and 344,901 controls of European ancestry. Further details are available in the original publication.

### MR and Bayesian colocalization analysis

All analyses were conducted using the TwoSampleMR package [26] in the R environment. In the primary models, gene expression in each immune cell subtype was defined as the exposure, with migraine and its subtypes as the outcomes. For genes with a single instrumental variable, the Wald ratio was used; for those with two or more, the random-effects inverse variance weighted (IVW) model served as the primary method [27]. To assess robustness against horizontal pleiotropy, we performed sensitivity analyses stratified by the number of SNPs: for genes with two SNPs, MR-Egger intercept test, weighted median, and Cochran’s Q test; for genes with three or more, MR-PRESSO (with outlier correction) and leave-one-out analyses were additionally conducted. For single-SNP instruments, only Wald ratio estimates were reported, as sensitivity analyses were not applicable. To control for multiple testing, within each outcome, MR tests were performed for all gene–cell type pairs across the 28 immune cell subtypes, and FDR correction was applied independently within each cell type, with statistical significance set at FDR-adjusted *p* < 0.05 [28].

Bayesian colocalization analysis was conducted using the coloc package in R [29] to determine whether gene expression and migraine (or its subtypes) share common causal genetic variants, thereby distinguishing spurious associations due to linkage disequilibrium. Default prior probabilities were used: p1 = 1 × 10^− 4^, p2 = 1 × 10^− 4^, and p12 = 1 × 10^− 5^.The posterior probabilities of five competing hypotheses were systematically evaluated: H0 (neither trait is associated with any genetic variant), H1 (only gene expression is associated), H2 (only the disease is associated), H3 (both are associated but driven by distinct causal variants), and H4 (both share the same causal variant). According to established criteria [30], PPH4 ≥ 0.8 was considered strong evidence of colocalization. Only immune cell gene targets meeting both criteria (P-FDR < 0.05 and PPH4 ≥ 0.8) were retained for further in-depth analysis.

### Genetic association analysis and validation of multiple effects across diseases

To evaluate the genetic correlation between migraine (and its subtypes) and psychiatric disorders (ANX, BIP, depression, OCD, and PTSD), the LDSC method was first employed to perform regression analyses, estimating the genetic correlation coefficient (rg) and its standard error, as well as heritability (h^2^). Additionally, the HDL method was applied to re-estimate the genetic correlations. A positive rg indicates shared genetic risk variants, whereas a negative rg suggests opposing genetic effects. Statistical significance was assessed using two-sided Z-tests (*p* < 0.05), and multiple testing correction was performed using FDR.

For cross-disease pleiotropy validation of immune cell gene targets, SNPs from positive targets were used as instrumental variables, and Mendelian randomization was applied to evaluate potential causal effects using the same MR approach as described above. Odds ratios (ORs) with 95% confidence intervals were calculated, and FDR correction was applied, with P-FDR < 0.05 considered statistically significant.

### Replication analysis

To validate the robustness of our prioritized targets, we applied the same scTWMR procedure to the genes identified in the preceding steps as having cross-disease pleiotropic effects, using independent datasets. Specifically, the immune-cell eQTL data from TenK10K were replaced with the OneK1K cohort (comprising 14 immune-cell subtypes), and the FinnGen summary statistics for migraine GWAS outcome data were replaced with the dataset from Wema A et al.

### Gene ontology, pathway enrichment analysis and protein-protein interaction

To further elucidate the biological functions and potential regulatory mechanisms associated with immune cell gene targets, this study performed systematic functional enrichment analysis and protein–protein interaction (PPI) network construction. For functional annotation, the R package clusterProfiler [31] and the SRplot online platform [32]were jointly used to conduct Gene Ontology (GO) annotation and KEGG pathway enrichment analysis. GO analysis systematically characterized the functional features of the target gene set from three perspectives: biological process, molecular function, and cellular component, and identified key signaling pathways and metabolic routes involved, with a significance threshold of *p* < 0.05 for initial screening. For protein interaction analysis, a PPI network was constructed using the STRING database (version 12.0 [33]); to explore functional synergies among proteins encoded by core genes. The interaction confidence threshold was set to a high level (combined score ≥ 0.7) [34], with the maximum number of first-shell interactors per seed protein limited to 10, while all other parameters were kept at the database defaults.

### Phenome-wide association study and known drug–gene interactions

To comprehensively assess the pleiotropic effects and potential safety profiles of prioritized immune cell gene targets, we conducted a phenome-wide association study (PheWAS) using the AstraZeneca PheWAS platform (https://azphewas.com/) [35], which integrates genomic data from approximately 500,000 UK Biobank participants across six ancestral groups, covering ~13,000 binary and ~5,000 continuous phenotypes. Both variant-level and gene-level association tests were performed, with statistical significance set at *p* < 5 × 10^− 8^ to mitigate false positives. For drug–gene interaction analysis, we queried DrugBank [36], ChEMBL [37], and DGIdb [38] to retrieve known interacting compounds and approved indications for the prioritized immune cell gene targets. These database queries identify pharmacological accessibility of the prioritized targets but do not establish therapeutic efficacy.

## Results

### Screening results of immune cell-specific gene tool variables

**Fig. 2:**
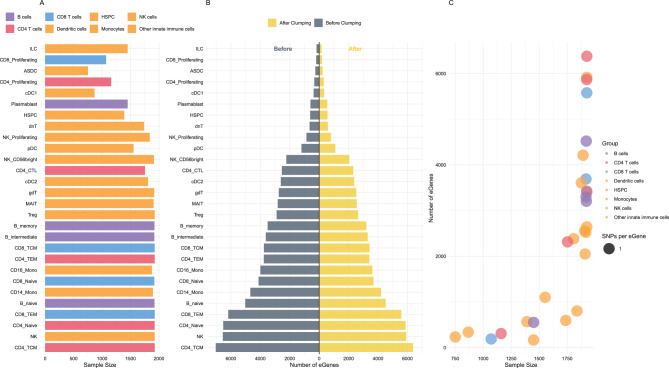
Sample sizes, cis-eQTL distribution, and instrumental variable characteristics of immune cell subtypes. (**A**) illustrates the total sample size distribution across 28 immune cell subtypes, with distinct colors differentiating eight major cell lineages: B cells, CD4+ T cells, CD8+ T cells, dendritic cells, HSPC, monocytes, NK cells, and other innate immune cells. (**B**) demonstrates the distribution of immune cell-specific cis-eQtls before and after clumping. CD4+TCM: CD4+ T central memory cells; NK: natural killer cells; CD4+Naive: CD4+ naïve T cells; CD8+TEM: CD8+ T effector memory cells; B_naive: naïve B cells; CD14+Mono: CD14+ monocytes; CD8+Naive: CD8+ naïve T cells; CD16+Mono: CD16+ monocytes; CD4+TEM: CD4+ T effector memory cells; CD8+TCM: CD8+ T central memory cells; B_intermediate: intermediate B cells; B_memory: memory B cells; Treg: regulatory T cells; MAIT: mucosal-associated invariant T cells; gdT: gamma delta T cells; cDC2: conventional dendritic cells type 2; CD4+CTL: CD4+ cytotoxic T cells; NK_CD56bright: CD56bright natural killer cells; pDC: plasmacytoid dendritic cells; NK_Proliferating: proliferating natural killer cells; dnT: double-negative T cells; HSPC: hematopoietic stem and progenitor cells; Plasmablast: plasmablasts; cDC1: conventional dendritic cells type 1; CD4+Proliferating: proliferating CD4+ T cells; ASDC: AXL+SIGLEC6+ dendritic cells; CD8+Proliferating: proliferating CD8+ T cells; ILC: innate lymphoid cells. (**C**) shows the proportion of gene-cell combinations retaining only a single SNP as the instrumental variable for each cell type in the final instrumental variable set

Based on 28 immune cell subtypes from the TenK10K cohort, the sample sizes in this study ranged from 751 to 1,925. The cells were classified into eight major groups: B cells, CD4+ T cells, CD8+ T cells, dendritic cells, HSPCs, monocytes, NK cells, and other innate immune cells. Among these, CD4+ TCM, NK, CD4+ Naive, and CD8+ TEM had the largest sample sizes, whereas ASDC, cDC1, and CD8+ Proliferating had relatively smaller sample sizes (Fig. 2A). In the cis-eQTL identification stage, a linkage disequilibrium threshold of r^2^ < 0.001 was used for clumping, and weak instrumental variables with an F-statistic < 10 were excluded. The number of eGenes varied across cell types, with CD4+ TCM having the highest count (7,031 before clumping; 6,379 after clumping) and CD8+ Proliferating the lowest (207 and 188, respectively). The distribution patterns before and after clumping were largely consistent, with CD4+ TCM and NK cells ranking as the top two (Fig. 2B). Ultimately, 87.3% to 95.9% of cell–gene combinations retained only one independent cis-eQTL as an instrumental variable (Fig. 2C).

### Immune cell gene targets for migraine

**Fig. 3:**
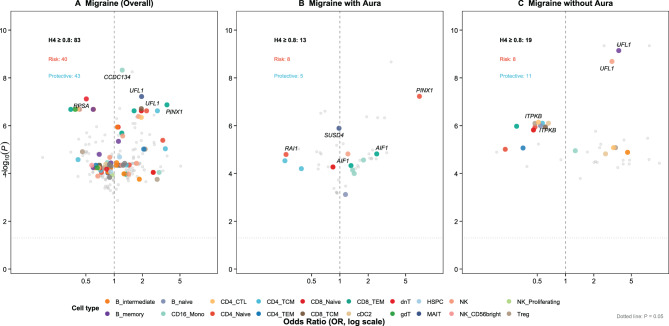
Single-cell transcriptome Mendelian randomization identifies immune cell–specific gene targets for migraine risk. Volcano plots for (**A**) overall migraine, (**B**) migraine with aura, and (**C**) migraine without aura. Each point represents a gene–immune cell pair, colored by immune cell type. The x‑axis shows the odds ratio (OR), and the y‑axis shows –log₁₀ (FDR‑adjusted P). The vertical dashed line indicates OR = 1 (protective left, risk right)

Based on scTWMR and colocalization analyses, a total of 83 significant immune cell gene targets (40 with risk effects and 43 with protective effects) were identified for overall migraine (Fig. 3A), with UFL1, PINX1, RPSA, and CDC131 as representative targets across CD4^+^ T cells, CD8^+^ T cells, monocytes, and NK cell subtypes. For migraine with aura, 13 significant targets (8 risk, 5 protective) were identified (Fig. 3B), including PINX1, SUSD4, and AIF1 in CD8^+^ T cells and monocytes. For migraine without aura, 19 significant targets (8 risk, 11 protective) were identified (Fig. 3C), with UFL1 and ITPKB as prominent targets across CD4^+^ T cells, CD8^+^ T cells, NK cells, and other subtypes. All significant results met the criteria of P-FDR < 0.05 and PP.H4 ≥ 0.8, indicating robust genetic colocalization evidence linking these specific immune cell gene targets to different migraine subtypes (Details of immune targets are provided in Table S1 in the Supplementary material).

### Genetic correlation between migraine and psychiatric disorders

**Fig. 4:**
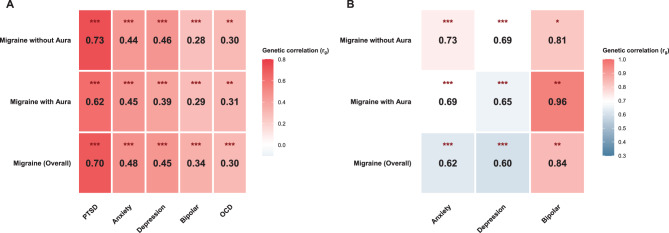
Genetic correlations between migraine subtypes and mental disorders estimated by (**A**) LDSC and (**B**) HDL regression methods. The numbers represent the magnitude of genetic correlations. *** *p* values after FDR correction < 0.001, ** *p* values after FDR correction < 0.01, * *p* values after FDR correction < 0.05. PTSD, post-traumatic stress Disorder; OCD, obsessive-compulsive disorder

LDSC analysis revealed that migraine and its subtypes exhibited significant positive genetic correlations with several psychiatric disorders (Fig. 4A). The strongest correlation was observed with PTSD (rg = 0.62–0.73), followed by ANX (rg = 0.44–0.48) and depression (rg = 0.39–0.46). Migraine also showed significant but weaker positive correlations with BIP (rg = 0.28–0.34) and OCD (rg = 0.30–0.31), all of which remained significant after FDR correction. HDL analysis further validated the substantial genetic overlap between migraine and depression, ANX, and BIP (rg ≈ 0.60–0.73) (Fig. 4B). Overall, migraine and its subtypes share a common genetic basis with common psychiatric disorders, including ANX, depression, PTSD, BIP, and OCD (Table S3-3).

### Validation of cross-disease multi-effectivity of immune cell gene targets

**Fig. 5:**
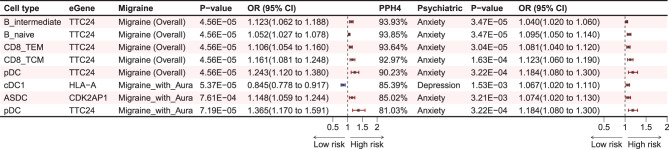
Validation of cross-disease pleiotropic effects of immune cell gene targets in migraine and psychiatric disorders. This forest plot displays the associations of celltypespecific genes with migraine (overall, with aura, or without aura) and psychiatric disorders (anxiety or depression). For each association, the cell type, gene, *p* value, odds ratio (OR) with its 95% confidence interval, and posterior probability (PPH4) are presented. The colorgradient bars at the bottom represent the OR scale, where blue indicates low risk (protective effect) and red indicates high risk (risk effect)

Across multiple immune cell types, several gene expression traits demonstrated significant associations with migraine (and its subtypes) as well as with anxiety or depression (Fig. 5). Specifically, TTC24 expression in B intermediate, B naive, CD8^+^ TCM, CD8^+^ TEM, and pDC cells was significantly associated with migraine (*p* = 4.56 × 10^− 5^, OR range = 1.052–1.243) and anxiety (*p* = 1.63 × 10^− 5^, OR range = 1.040–1.184). Subtype analysis further revealed that TTC24 expression in pDC cells was also associated with migraine with aura (OR = 1.365, *p* = 7.19 × 10^− 5^) and anxiety (OR = 1.184, *p* = 3.22 × 10^− 4^). CDK2AP1 expression in ASDC cells similarly exhibited risk effects for both migraine with aura (OR = 1.148, *p* = 7.61 × 10^− 4^) and anxiety (OR = 1.074, *p* = 3.21 × 10^− 3^). In contrast, HLA-A expression in cDC1 cells displayed a protective effect against migraine with aura (OR = 0.845, *p* = 5.37 × 10^− 5^) while conferring an increased risk for depression (OR = 1.067, *p* = 1.53 × 10^− 3^). The posterior probability of hypothesis 4 (PPH4) values for all aforementioned associations were ≥ 80% (range: 81.03%–93.93%), supporting the robustness of these findings and further corroborating the cross-disorder pleiotropic effects of immune-related genes in migraine, its subtypes, and psychiatric disorders.

### Replication of prioritized targets using independent datasets

Replication analyses revealed (Table S8-9) that CDK2AP1 demonstrated consistent effects in ASDC cells, HLA-A likewise exhibited consistent effects in conventional dendritic cells (including cDC1 cell), and TTC24 showed significant associations across multiple T‑cell subsets. These findings further support the robustness of our prioritized targets; nevertheless, replication in additional independent cohorts remains essential.

### Gene ontology, pathway enrichment analysis and protein-protein interaction

**Fig. 6:**
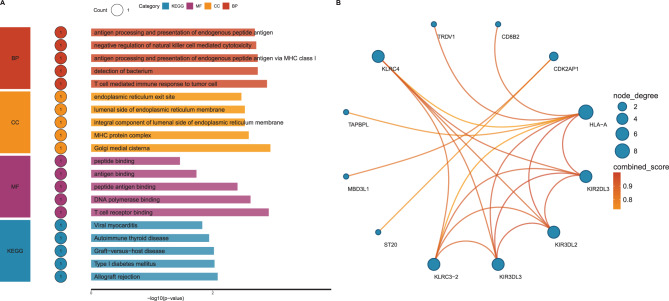
Functional enrichment and protein-protein interaction network analyses of the identified potential immune cell-related gene targets. (**A**) Gene ontology (GO) and KEGG pathway enrichment analyses, displaying the top 5 significantly enriched terms per category (see complete results in table S2–S5). (**B**) Protein-protein interaction (PPI) network construted using the STRING database. BP: biological Process; MF: molecular Function; CC: cellular component

Enrichment analysis revealed that the identified potential immune cell gene targets were significantly enriched in immune pathways such as graft-versus-host disease, type I diabetes mellitus, and autoimmune thyroid disease, as well as in Gene Ontology (GO) terms including T cell receptor binding, antigen processing and presentation, and MHC protein complex (Fig. 6A). Protein-protein interaction (PPI) network analysis further identified HLA-A, KIR2DL3, KIR3DL2, and KLRC4 as hub genes (Fig. 6B). These findings suggest that the complex regulatory network involving immune cell-related genes may participate in the pathophysiological processes underlying migraine and psychiatric disorders.

### PheWAS and known drug–gene interactions

PheWAS analysis revealed no significant associations between the prioritized immune cell gene targets and adverse clinical outcomes (Supplementary Materials Figure), suggesting favorable safety profiles for these targets. Database queries identified multiple approved drugs with known interactions with HLA-A that are used in the treatment of epilepsy, depression, or migraine (Table 1). Among them, antiepileptics (carbamazepine, levetiracetam, zonisamide) have been investigated for migraine prophylaxis, whereas tricyclic antidepressants (clomipramine, trimipramine, desipramine, doxepin) are standard treatments for depression, anxiety, and insomnia. These observations identify pharmacological accessibility of the prioritized genetic target, but do not demonstrate that the clinical effects of these drugs are mediated through HLA-A or the cDC1 axis, nor do they establish therapeutic efficacy. Therefore, these findings are hypothesis-generating and require further investigation.

**Table 1:** Known drug-gene interactions of immune cell gene targets

Gene	Cell type	Drug	Actions	Modality	Trial status	Indication	Max phase
HLA-A	cDC1	Carbamazepine	Inhibitor	Small molecule	Approved	A drug used for treating various types of epileptic seizures and mood stabilizers	Phase 4
		Levetiracetam	Inhibitor	Small molecule	Approved	A medication used to treat seizures.	Phase 4
		Zonisamide	Inhibitor	Small molecule	Approved	A medication used to treat some seizure disorders.	Phase 4
		Clomipramine	Inhibitor,Antagonist	Small molecule	Approved	Tricyclic antidepressants	Phase 4
		Trimipramine	Inhibitor,Antagonist	Small molecule	Approved	Tricyclic antidepressants	Phase 4
		Desipramine	Inhibitor,Antagonist	Small molecule	Approved	Tricyclic antidepressants	Phase 4
		Doxepin	Inhibitor,Antagonist	Small molecule	Approved	Antidepressive Agents Indicated for Depression	Phase 4

## Discussion

In this study, a systematic analysis of 28 immune cell subtypes was conducted by integrating scTWMR, colocalization analysis, genetic correlation analysis, and cross-disease pleiotropy validation. A total of 83 immune cell-related gene targets associated with migraine were identified, among which 6 specific targets were also significantly associated with anxiety (ANX), and 1 with depression. These findings provide insights into the potential molecular mechanisms underlying migraine and psychiatric disorders from an immunogenetic perspective, and may offer preliminary target clues for investigating known drug–gene interactions and future individualized immunotherapeutic strategies.

### Shared immune cell gene targets between migraine and psychiatric disorders

LDSC and HDL analyses confirmed significant positive genetic correlations between migraine and ANX and depression (rg = 0.39–0.73), consistent with previous cross-trait studies [39–41]. The present study further identified several genes shared between migraine and psychiatric disorders, with effects showing high immune cell type specificity. In the genetic overlap between migraine and ANX, TTC24 exhibited consistent cross-disorder risk effects across multiple immune cell subsets (B_intermediate, B_naive, CD8^+^ TCM, CD8^+^ TEM), and notably, its expression in pDCs was significantly associated with both migraine (including its aura subtype) and ANX. This suggests that TTC24 may serve as a pan-immune cell gene target linking migraine and ANX, with pDCs potentially representing a key immune cell type mediating the genetic association between migraine with aura and ANX. TTC24 encodes a tetrapeptide repeat protein 24, which may be involved in the regulation of immune system function and cell cycle regulation. Immunological studies have reported elevated B cell subsets and B-cell activating factor (BAFF) levels during migraine attacks, as well as significant alterations in CD8^+^ T cell subsets associated with disease severity [42]. Single-cell studies have confirmed increased T cell abundance and an activated phenotype in migraine patients [43, 44], and MR has suggested a causal link between CD8^+^ T cells and anxiety disorder [45]. Furthermore, CDK2AP1 exhibited stable cross-disorder risk associations in ASDCs, with its expression level positively correlated with various immune cell infiltrates and involved in immune processes such as Th1/Th2 cell differentiation [46]. ASDCs represent a recently identified dendritic cell subset with potent T cell activation capacity. This study is the first to link TTC24 and CDK2AP1 to specific immune cell subsets, suggesting that they may influence neuroinflammation, synaptic transmission, and anxiety-related neural circuits through mechanisms involving immune modulation and neurotransmitter secretion, thereby contributing to the genetic susceptibility shared between migraine and ANX. These findings provide new entry points for subsequent functional validation and mechanistic studies.

Notably, HLA-A in cDC1s showed directionally discordant pleiotropy: it was associated with reduced risk of migraine with aura but increased risk of depression, suggesting that certain genes may exert opposite functional effects in different disease contexts. HLA-A encodes a class I major histocompatibility complex molecule that mediates cellular immune responses by presenting antigenic peptides to CD8^+^ T cells and participates in immune surveillance [47, 48]. cDC1s are a dendritic cell subset with high cross-presentation capacity, activating CD8^+^ T cells via MHC-I and promoting Th1 responses [49]. In migraine with aura, cortical spreading depression can activate meningeal dendritic cells, which are in close contact with TRPV1^+^ nociceptor axons and may be involved in headache regulation [50]. In depression, dendritic cells exhibit sex-specific alterations in cytokine production, and increased frequency of IL-23^+^ cDCs is associated with depression severity [51]. These findings suggest that migraine and psychiatric disorders may share overlapping immunogenetic mechanisms. The opposing effects of the HLA-A/cDC1 axis on the two diseases further reveal the complexity of genetic pleiotropy.

### Known drug–gene interactions of prioritized immune cell gene targets

For the HLA-A/cDC1 target, database queries identified approved antiepileptic drugs (carbamazepine, levetiracetam, zonisamide) and tricyclic antidepressants (clomipramine, doxepin, trimipramine, desipramine) with known interactions with HLA-A. Carbamazepine may benefit patients with epilepsy and migraine [52, 53]. Levetiracetam reduces headache frequency and shows greater efficacy in migraine with aura; however, 8–16% of users may experience worsening of depressive and anxiety symptoms, particularly those with a history of mood disorders [54]. Zonisamide shows no significant difference in efficacy from topiramate; approximately 7% of patients at high doses may develop mood disorders and induce depression [55]. Regarding tricyclic antidepressants, doxepin and desipramine have shown positive results in small trials, whereas clomipramine is not recommended for migraine prophylaxis due to poor efficacy and a high rate of adverse effects [56]. The directionally opposing genetic effects of the HLA-A/cDC1 target on migraine with aura (protective) and depression (risk) offer a testable hypothesis for understanding the discordant clinical profiles of these drug classes across the two conditions. For other prioritized immune cell gene targets, although no known drug interactions were identified, PheWAS analysis indicated favorable safety profiles, providing preliminary support for future target-specific therapeutic exploration. All drug-related findings presented here are hypothesis-generating and should not be interpreted as evidence of clinical utility or therapeutic repurposing.

### Limitations

This study has several limitations. First, the study population was predominantly of European ancestry, and the findings require validation in other populations. Second, the sample size of the immune cell eQTL dataset was limited, which restricted the number of detectable genes and eQTLs and also resulted in most genes being associated with only a single eQTL, precluding sensitivity analyses to correct for horizontal pleiotropy [17]; colocalization analysis could not rule out the possibility that a single causal variant exerts pleiotropic effects on multiple neighboring genes [57]. Third, although we replicated the main findings in independent eQTL and GWAS datasets, differences in single‑cell RNA‑sequencing experimental protocols may lead to variability in results, and the greater granularity of cell type classification in the discovery dataset compared with the validation dataset may limit their direct comparability. Fourth, the drug-gene interactions derived from the database have not been experimentally verified. Therefore, the clinical efficacy of the identified drugs cannot be clearly attributed to the gene targets prioritized in this study. Further functional validation is still required in the future.

## Conclusion

In summary, through systematic genetic analysis of 28 immune cell subtypes, this study identified 83 immune cell gene targets associated with migraine. Among these, 6 showed shared associations with anxiety and 1 with depression, highlighting the involvement of immune-cell-specific genes in the genetic landscape shared between migraine and psychiatric disorders. Genetic correlation analyses further confirmed a shared genetic basis between migraine and several psychiatric disorders, including anxiety, depression, PTSD, bipolar disorder, and obsessive-compulsive disorder. Notably, antiepileptic drugs and tricyclic antidepressants with known interactions with HLA-A offer a testable hypothesis for understanding the discordant clinical profiles of these drug classes across migraine with aura and depression, providing a preliminary immunogenetic basis for future personalized treatment strategies in patients with co-occurring conditions. Future research should conduct prospective cohort studies to validate whether HLA-A genotype-informed drug selection improves clinical outcomes in comorbid patients. In addition, functional validation and drug screening for the remaining prioritized immune cell gene targets warrant further investigation.

## Electronic supplementary material

Below is the link to the electronic supplementary material.

Supplementary material 1

Supplementary material 2

## Data Availability

Data supporting the findings of this study are available from the article/Supplementary material.
