# Supplementary material for: Migraine immune cell gene targets and their relationship to psychiatric disorders
Source: J Headache Pain. 2026 Jul 25;27(1):205. doi: 10.1186/s10194-026-02465-1 (PMC13464352; doi:10.1186/s10194-026-02465-1)
Supplement: Supplementary file 1 [file 10194_2026_2465_MOESM1_ESM.docx]

***Supplementary Materials***

Fig A: PheWAS analysis of CDK2AP1 and TTC24

Note: CDK2AP1 and TTC24 were not found to be significantly associated with binary (left panel) or continuous adverse phenotype (right panel). PheWAS, phenome-wide association study。

Fig B:PheWAS analysis of HLA-A

Note: HLA-A were not found to be significantly associated with binary (left panel) or continuous adverse phenotype
